# Approximating Intermediate Feature Maps of Self-Supervised Convolution Neural Network to Learn Hard Positive Representations in Chest Radiography

**DOI:** 10.1007/s10278-024-01032-x

**Published:** 2024-02-21

**Authors:** Kyungjin Cho, Ki Duk Kim, Jiheon Jeong, Yujin Nam, Jeeyoung Kim, Changyong Choi, Soyoung Lee, Gil-Sun Hong, Joon Beom Seo, Namkug Kim

**Affiliations:** 1 Department of Bioengineering, Asan Medical Institute of Convergence Science and Technology, Asan Medical Center, 88 Olympic-Ro 43-Gil Songpa-Gu, Seoul, 05505 South Korea; 2 Department of Convergence Medicine, University of Ulsan College of Medicine, Asan Medical Center, 88 Olympic-Ro 43-Gil Songpa-Gu, Seoul, 05505 South Korea; 3 Department of Radiology and Research Institute of Radiology, Asan Medical Center, University of Ulsan College of Medicine, Seoul, Republic of Korea

**Keywords:** Chest radiograph (CXR), Contrastive learning, Hard negative representation, Hard positive representation, Self-supervised learning

## Abstract

Recent advances in contrastive learning have significantly improved the performance of deep learning models. In contrastive learning of medical images, dealing with positive representation is sometimes difficult because some strong augmentation techniques can disrupt contrastive learning owing to the subtle differences between other standardized CXRs compared to augmented positive pairs; therefore, additional efforts are required. In this study, we propose intermediate feature approximation (IFA) loss, which improves the performance of contrastive convolutional neural networks by focusing more on positive representations of CXRs without additional augmentations. The IFA loss encourages the feature maps of a query image and its positive pair to resemble each other by maximizing the cosine similarity between the intermediate feature outputs of the original data and the positive pairs. Therefore, we used the InfoNCE loss, which is commonly used loss to address negative representations, and the IFA loss, which addresses positive representations, together to improve the contrastive network. We evaluated the performance of the network using various downstream tasks, including classification, object detection, and a generative adversarial network (GAN) inversion task. The downstream task results demonstrated that IFA loss can improve the performance of effectively overcoming data imbalance and data scarcity; furthermore, it can serve as a perceptual loss encoder for GAN inversion. In addition, we have made our model publicly available to facilitate access and encourage further research and collaboration in the field.

**Supplementary Information:**

The online version contains supplementary material available at 10.1007/s10278-024-01032-x.

## Introduction

Chest radiograph (CXR) is not only one of the most commonly performed radiological examinations in routine clinical practice but also an important examination owing to the useful information it can provide to clinicians [1, 2]. Although CXRs are frequently used and relatively easy to obtain, their interpretation requires expertise. Considering the labor and cost involved in interpreting the vast number of radiological images, deep learning has emerged as an assistant for CXR interpretation [3, 4]. Deep learning methods can assist radiologists in reading CXRs with abnormality classification [5], detection [6], and segmentation [7]. However, training deep learning models on medical images poses several challenges, such as privacy issues, inaccessibility, and high costs.

Self-supervised learning (SSL) has emerged as a solution to these challenges in medicine [8]. SSL is an unsupervised pretraining method that learns representations from self-defined tasks. Researchers can reduce the burden of using expensive labels for medical images because a network learns representations using self-defined labels in SSL. A deep learning network can learn better visual representations of medical images by solving jigsaw puzzles [9, 10], Rubik’s cubes of medical images [11–13], and restoring corrupted images [14, 15]. The contrastive learning method is one of the most powerful SSL methods for learning visual representations. Some approaches have been adopted to improve the performance of deep learning networks using contrastive learning methods. For example, one study showed that pretraining with contrastive learning on medical images such as a dermatology dataset or CXR dataset improves the performance of a deep learning network on classification task [16], and Ghesu et al. trained a contrastive network on 100 million medical images and showed improved performance on classification and object detection of CXR, chest CT, and brain MRI [17]. Cho et al. trained a contrastive network on 4.8 million CXR images and made the pretrained weights publicly available [18].

**Fig. 1:**
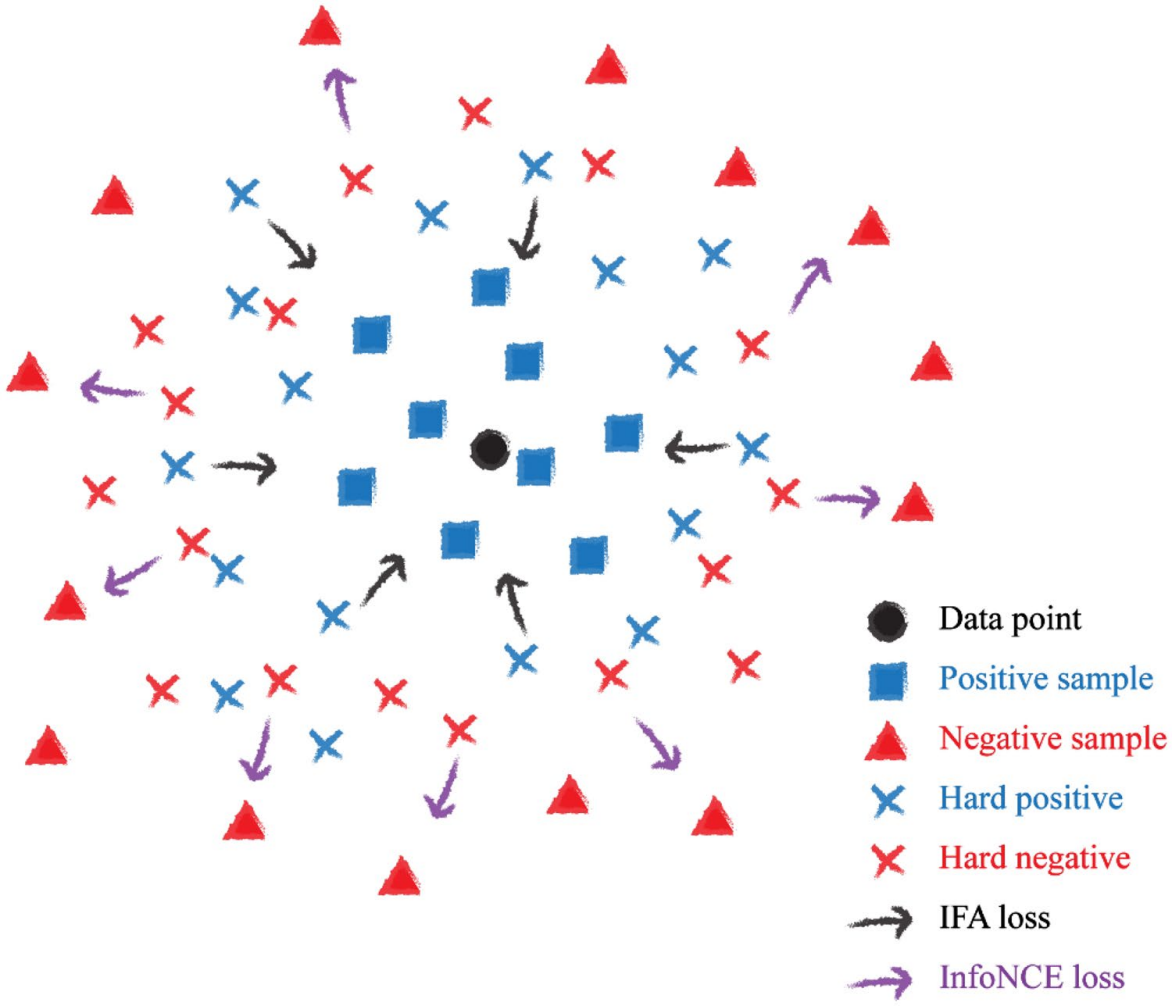
The diagram of negative sample, positive sample, and original data point in the latent space. Some positive samples that are located farther from the query (data point) than hard negative samples can be considered as “hard positive” samples

Contrastive learning trains a network by placing similar images (positive pairs) closer together and different images (negative pairs) further apart in the latent space. Typically, contrastive learning uses multiple views of the original image created by image augmentation as positive pairs and the other images as negative pairs [19]. Although contrastive learning is a powerful method for learning visual representations, it also suffers from some challenges, such as “hard negatives” [20–22]. Hard negatives refer to the negative pairs that are closer to the original data point than the positive pair in latent space, making them a “hard” representation. Positive pairs that are further away from the original data point than negative pairs can be referred to as “hard positives.” Therefore, addressing these hard representations is crucial in training contrastive learning. Figure 1 depicts the relationship between the hard positives and hard negatives in the latent space.

Hard representations are prevalent in a medical image dataset owing to the standardized acquisition protocols. Therefore, common data augmentation techniques used in contrastive learning may result in variations that are larger than those present in the negative samples. For example, anatomical variations, positions, and breath-hold levels can vary more than strong augmentations, resulting in some negative samples being more similar to the data point than the positive pairs generated with these strong augmentations. To deal with the problem of hard negatives, researchers use large memory banks of negative samples [23, 24] or sample and mix hard negative samples [20–22]. However, most contrastive learning studies on medical images focus mainly on hard negatives and address the positive pair only to the extent of finding good augmentation combinations [23, 25]. Therefore, additional efforts beyond augmentations can help a contrastive network train positive representations effectively in medical imaging. Recently, a study group reported that addressing hard positives by weighting the cosine similarity score of linear projections of feature vectors extracted from positive pairs can improve medical image segmentation [26].

In this study, we propose intermediate feature approximation (IFA) loss to deal with the hard positives and improve the baseline of the pretrained contrastive network. We expect IFA loss to pull positive pairs closer to the original point by approximating the intermediate feature maps of positive pairs. Therefore, a contrastive learning network can set decision boundaries between negative and positive pairs a little easier. We demonstrated the results of the IFA loss fine-tuned contrastive network with various downstream tasks.

## Materials and Methods

This retrospective study was conducted according to the principles of the Declaration of Helsinki and according to current scientific guidelines. The study protocol was approved by the Institutional Review Board Committee (IRB) of tertiary hospital. The requirement for written informed consent was waived by the IRB because the data were analyzed retrospectively and anonymously. The detailed materials and methods of the study are provided in this section.

### Training Hard Positive Representations to Learn Better Visual Representation of CXR

A total of 4.8 M CXR images composed of 3.6 M adult CXR images collected from 2011 to 2018 and 1.2 M pediatric CXR images collected from 1997 to 2018 were used to learn visual representations of CXR [18]. CXRs have been obtained retrospectively from a South Korean tertiary hospital. Only the posterior-anterior view images of CXRs were included in this study.

In this study, we propose intermediate feature approximation (IFA) loss, aiming to increase the similarity between the intermediate feature maps of positive pairs. IFA loss was calculated as follows:

1 $${f}_{\theta }\left(Query\right)=Q$$

2 $${f}_{\theta }\left(Positive\;pair\right)=P$$

3 $$\Vert 1-{\text{cos}}(Q, P)\Vert$$

where *f*_*θ*_ denotes the contrastive network before the target approximation output layer; *Q* denotes the intermediate feature tensor output of the query image after the target approximation layer; *P* denotes the intermediate feature tensor output of the positive pair image after the target approximation layer. The cosine similarity between the tensor outputs was calculated and subtracted with the tensors composed of 1 with the same shape as the cosine similarity matrix. IFA loss then maximizes the cosine similarity between the *Q* and *P*. However, training contrastive networks only with hard representations from scratch may lead to their failure to converge [22]. Therefore, we used IFA loss as a supportive function to improve the existing pretrained network.

**Fig. 2:**
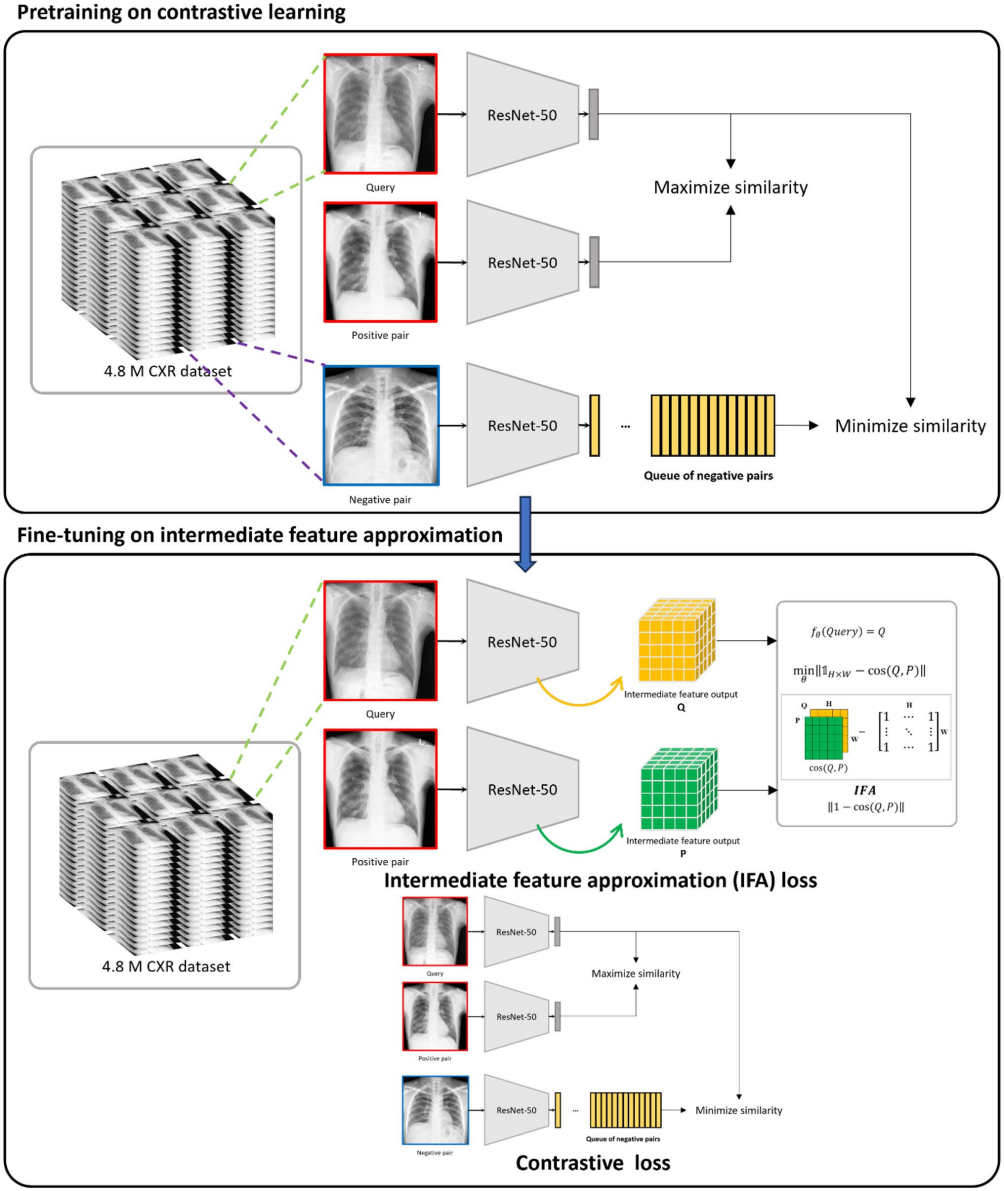
Overall workflow of upstream fine-tuning of intermediate feature approximation (IFA) loss. A ResNet-50 model was first pretrained on 4.8 million CXR datasets using the MoCo v2 or SimCLR method. After contrastive pretraining, the pretrained ResNet-50 was fine-tuned jointly using the IFA loss and the contrastive loss used in the initial pretraining

Therefore, we first trained a self-supervised contrastive network with unlabeled images based on MoCo v2 [23]. InfoNCE loss [27] was used to maximize the similarity between positive pairs and minimize the similarity between negative pairs for the MoCo v2 baseline. A self-supervised contrastive network based on SimCLR [25] was also trained to see if the performance improvement of IFA loss could be generalized to other contrastive networks. The NT-Xent loss was used for SimCLR. When IFA loss was used to fine-tune the pretrained network, it was used in conjunction with InfoNCE loss or NT-Xent loss, which was used to train the pretrained network initially. The graphical summary of the methods is shown in Fig. 2.

The ResNet-50 [28] architecture, one of the most commonly used CNN architectures, has been used throughout the experiment. The ResNet-50 architecture consists of an initial 7 × 7 convolutional layer and four bottleneck blocks, which consist of multiple 3 × 3 convolutional layers surrounded by 1 × 1 bottleneck convolutions back and forth. Although the IFA loss can be applied to any of the 50 intermediate feature outputs of ResNet-50, we experimented with four intermediate feature outputs after each of the four bottleneck blocks to determine the optimal level of approximation to improve network performance, considering the limited resources and time. Accordingly, the performance of approximating the outputs of residual blocks 1, 2, 3, and 4 outputs was compared.

### Evaluation via Various Downstream Target Tasks

Several downstream tasks relevant to medical imaging were experimented with to evaluate our pretrained models. First, image classification tasks were evaluated. An image classification task, which requires using comprehensive features from an image, is a key application of deep learning in medical imaging. A multiclass classification task using a private dataset [29], a multilabel classification task using the CheXpert [30] dataset, and a pediatric pneumonia classification task [31] were conducted for classification tasks.

In the CXR 6-class classification task [29], the ability of each pretrained model to handle class imbalance, which is common in clinical situations, was conducted to classify normal, nodule, consolidation, interstitial opacity, pleural effusion, and pneumothorax from CXR, which are CT confirmed data labels. Severe data imbalance was established in the initial setting, with the most prevalent disease class having 1540 images and the least prevalent disease class 280 images. In addition to the initial setting, undersampled data with the least common number of images was also set up to compare the model performance in the fair but limited amount of data settings. Finally, a modified dataset was set in which the amount of data was adjusted based on the difficulty of each class in the dataset. A balanced validation and test dataset was created because the performance can be overestimated if a similar prevalence to the train dataset is applied to them. In addition, the validation and test datasets were kept consistent across all experiments to ensure a fair comparison between each training dataset. Datasets for CXR 6-class classification are summarized in Table 1.

**Table 1:** Datasets used in CXR 6-class classification

	Normal	Nodule	Consolidation	Interstitial opacity	Pleural effusion	Pneumothorax
Full	2515	888	1484	224	1308	274
Undersampled	224	224	224	224	224	224
Modified	336	560	560	448	224	224
Validation	28	28	28	28	28	28
Test	28	28	28	28	28	28

The same number of images for each class was sampled for the undersampled dataset. Normal, nodule, and consolidation were additionally sampled for the modified dataset, while the interstitial opacity images were simply duplicated because no additional data was available for interstitial opacity

In the CheXpert multilabel classification task, multiple data fraction stress tests were conducted using fractions of 1%, 10%, 50%, and 100% to evaluate the performance of the model under varying levels of data availability, simulating the conditions commonly encountered in real-world research settings. Fine-tuning experiments on small data fractions were repeated 10 times using different random samples to ensure the reproducibility of the data stress test. A common unseen test dataset was used in all experiments to ensure a fair comparison. Data fractions for CheXpert are summarized in Table 2.

**Table 2:** Data fractions used in CheXpert multilabel classification

	1%	10%	50%	100%
Train	1787	17,873	89,366	178,732
Validation	446	4468	22,341	44,682
Test	234	234	234	234

To ensure the reproducibility of the data stress test, fine-tuning experiments on small data fractions of data were repeated 10 times using different random samples. All data was randomly sampled, and all CheXpert frontal view data was used

A pediatric pneumonia classification task was conducted because a considerable amount of pediatric CXRs were used in our pretraining task. In this study, a three-class classification task of distinguishing between normal, bacterial pneumonia, and viral pneumonia of pediatric CXR was evaluated. We randomly split the public training dataset into train and validation datasets at a ratio of 9:1. Offline augmentations was then used to balance the number of images among classes using rotation at the range of 10° and zooming range of 20%. The test dataset was kept the same as the public dataset. Datasets for pediatric pneumonia classification task are summarized in Table 3.

**Table 3:** Datasets used in pediatric pneumonia classification

	Normal	Bacterial pneumonia	Viral pneumonia
Train	2288	2288	2288
Validation	250	250	250
Test	234	242	148

The SIIM-FISABI-RSNA COVID-19 object detection [32, 33] was experimented with to evaluate the object detection performance of the proposed network. The Faster R-CNN [34] architecture with R50-dilated-C5 and R50-C4 was set as the object detection header. ResNet-50 was used as the backbone encoder for both architectures, and results were compared. The competition was designed to incorporate a single mAP score for both classification and object detection tasks. Furthermore, the scores we evaluated in this study could not be accurately obtained because the test datasets were hidden, and scoring was only possible through the submission process. The ground truth labels for the test datasets were not publicly available, only the trains were. Therefore, we randomly split the public training dataset into train, validation, and test datasets. Datasets for the COVID-19 lung opacity object detection task are summarized in Table 4.

**Table 4:** Datasets used in COVID-19 lung opacity object detection

	Negative	Lung opacity
Train	1388	3435
Validation	174	429
Test	174	430

Finally, our pretrained network was used to calculate medical perceptual loss [35] to assess its performance in capturing and representing relevant medical features. Projecting an original seed image into a latent feature using generative adversarial network (GAN) inversion was experimented with normal CXRs from the VinDr-CXR Chest X-ray Abnormalities Detection dataset [36]. The backbone for GAN inversion in our study was a style-based generative adversarial network [37]. The embedded latent vector was optimized to minimize the difference between the original and reconstructed image, using a combination of a pixel-wise mean squared error (MSE) loss and a perceptual loss [38]. VGG-16, ResNet-50 with supervised learning on ImageNet, ResNet-50 with MoCo v2 on ImageNet, ResNet-50 with SimCLR, CheSS (MoCo v2), and IFA loss combined with SimCLR and CheSS were used to calculate the perceptual loss and compared.

All datasets except the private dataset used in the multiclass classification were collected in institutions other than the upstream dataset, and the data used in the multiclass classification were not included in the upstream dataset. All datasets used to evaluate the downstream performance, except our private datasets, are publicly available online. The fine-tuning performance of each pretrained weight was compared in each downstream task.

### Statistical Analysis

Performance on the six-class classification task was evaluated by accuracies. The Stuart–Maxwell test [39, 40] for marginal homogeneity was performed to compare the performance of each model with the best-performance model.

Performance on CheXpert was evaluated by the area under the receiver operating characteristic curves (AUCs) because it is the standard performance metric for CheXpert. DeLong’s test for pairwise comparison of receiver operating characteristic (ROC) curves was performed to compare the performance of each model with the InfoNCE + IFA pretrained model. Means and standard deviations (SDs) of AUCs were calculated in the multiple data fraction stress test of CheXpert. Paired *t*-tests were performed to compare the performance of each model with the best performance model.

Performance on pediatric pneumonia classification tasks was evaluated by accuracy, precision, recall, and F1 scores. The Stuart–Maxwell test for marginal homogeneity was performed to compare the performance of each model with the best performance model.

Performance on COVID-19 lung opacity object detection was assessed using the mean of the average precision (mAP) at two different thresholds, mAP50 and mAP_COCO_ (0.5, [0.5:0.95]). The average precision (AP) is calculated as the mean of the precision at each threshold, and mAP is calculated as the mean of the AP of all data. Therefore, a null hypothesis test for lung opacity object detection was not required, given the large degree of freedom (i.e., all *p* values were < 0.001).

Performance on GAN inversion was evaluated using peak signal-to-noise ratio (PSNR), structural similarity index measure (SSIM), root mean squared error (RMSE), and learned perceptual image patch similarity (LPIPS). Paired *t*-tests were performed to compare the performance of each model with the best performance model.

All statistical analyses were performed using the R version of 4.2.1. Statistical significance was set at a two-sided *p* value of < 0.05.

## Results

### Implementation Details of Study Experiments

Our models were implemented with the PyTorch framework of 1.8.0. Training the upstream model required ~ 8 w. The implementation details are described as follows:

1. Preprocessing: All CXR images were resized into 512 × 512 pixels by linear interpolation. Next, to alleviate the high intensity of L/R markers in CXR images, we limited the maximum pixel value of the CXR images to the top 1% pixel of each CXR image [41].
2. Augmentation: To learn a more robust CXR representation of the model, strong but clinically viable extent data augmentation was performed using many transformations: *ShiftScaleRotate*, *MedianBlur*, *MotionBlur*, *Sharpen*, *Cutout*, *OpticalDistortion*, *RandBrightness*, *RandContrast*, *GaussNoise*, and *MultiplicativeNoise*.
3. Network comparison: One of the most widely used networks, ResNet-50 [28], was employed throughout this study. Our proposed IFA loss was compared with several other methods: random initialization without pretraining (Scratch), ImageNet pretrained with supervised learning (ImageNet-1 K), ImageNet pretrained with MoCo v2 (MoCo-ImageNet), SimCLR pretrained with CXR dataset (CXR-SimCLR), and MoCo v2 pretrained with CXR dataset (CheSS). IFA loss was additionally applied to the CXR-SimCLR model and CheSS model.
4. Training setting: The batch size of all experiments was set to the maximum for 8 GPUs memory (Tesla V100). In upstream, the batch size of 256 was used. The network was initialized by a uniform Xavier initialization and trained using an SGD optimizer with a learning rate of 1e − 5 using a weight decay of 1e − 4. The learning rate was reduced by the cosine learning rate schedule. No data leakage occurred between the train, valid, and test datasets in any downstream task. The entire model was fine-tuned in all downstream tasks. The implementation details for each downstream task are described in Supplementary file 1.

### Finding the Best Level of Feature Approximation Through the CheXpert Ablation Study

An ablation study was performed using the CheXpert dataset, which serves as a benchmark dataset for medical image classification, to determine the optimal layer for feature approximation. Table 5 presents the results of the ablation study. The models pretrained with IFA loss performed well compared to existing transferable weights, although they did not reach statistical significance in some classes. When IFA loss was combined with the SimCLR pretrained model, the overall performance increase was observed without statistical significance when IFA loss was used. When IFA loss was combined with the MoCo v2 pretrained model (CheSS), the overall performance increase was observed, and a statistically significant increase in edema (*p* < 0.001) and pleural effusion (*p* = 0.008) images was observed.

**Table 5:** AUCs of each pretrained model were experimented on the CheXpert dataset

	Atelectasis	Cardiomegaly	Consolidation	Edema	Pleural effusion	Mean AUC
Scratch	0.788^**^	0.725	**0.900**	0.813^***^	0.815^***^	0.808
ImageNet-1 K	0.815^*^	**0.805**	0.840^**^	0.896	**0.918**	0.855
MoCo-ImageNet	0.806^**^	0.800	0.852^*^	0.897	0.912	0.853
CXR-SimCLR	0.818^*^	0.797	0.864	0.888	0.903	0.854
CheSS (CXR-MoCo)	0.849	0.774	0.898	0.832^***^	0.869^**^	0.844
SimCLR + IFA	0.839	0.803	0.880	0.893	0.909	0.865
MoCo + IFA	**0.857**	0.785	0.899	**0.913**	0.905	**0.872**
Block 1	0.853	**0.791**	0.894	0.911	**0.908**	0.871
Block 2	0.844	0.787	0.878^*^	0.896^*^	0.901	0.861
Block 3 (ours)	0.857	0.785	0.899	0.913	0.905	**0.872**
Block 4	**0.858**	0.772	**0.902**	**0.915**	0.901	0.869

The bold text indicates the best performance

DeLong’s test was conducted for pairwise comparisons of each ROC

^*^p < 0.05; ^**^p < 0.01; ^***^p < 0.001

In the ablation study to find the best level of feature approximation, no statistically significant performance difference was observed between the levels of feature approximation output, except for the consolidation (*p* = 0.012) and edema (*p* = 0.031) outputs after residual block 2. We selected the best-performing model (block 3) for the other downstream tasks.

curve compared with the MoCo + IFA (block 3) model. *ROC*, receiver operating characteristics curve; *AUC*, area under receiver operating characteristics curve.

### Classification Performance Evaluation

Table 6 presents the results of all experiments conducted. IFA loss combined models showed significantly better performance in overcoming data imbalance compared with other commonly used pretrained models. When combined with IFA loss, the model performance was significantly improved in both SimCLR and CheSS in full dataset (*p* < 0.001, SimCLR; *p* = 0.004, CheSS) and modified dataset (*p* < 0.001, SimCLR; *p* = 0.013, CheSS). In addition, the model performance was significantly improved in SimCLR in undersampled dataset (*p* < 0.001).

**Table 6:** Accuracies of six-class classification model with multiple data imbalance simulations

	Full	Undersampled	Modified
Scratch	0.435^***^	0.357^***^	0.417^***^
ImageNet-1 K	0.440^***^	0.179^***^	0.262^***^
MoCo-ImageNet	0.577^***^	0.560^**^	0.464^***^
CXR-SimCLR	0.440^***^	0.351^***^	0.369^***^
CheSS (CXR-MoCo)	0.560^**^	**0.595**	0.512^*^
SimCLR + IFA	0.452^***^	0.405^***^	0.381^***^
MoCo + IFA	**0.637**	0.590	**0.589**

The bold text indicates the best performance

Stuart–Maxwell test was conducted to compare scratch (randomly initialized), ImageNet, MoCo-ImageNet, CheSS, and our pretrained (intermediate feature approximation loss) models

^*^p < 0.05; ^**^p < 0.01; ^***^p < 0.001

Second, a multilabel classification task of CheXpert [30] was conducted. Table 7 presents the results of all experiments conducted. IFA loss combined models showed significantly better performance in overcoming data shortage compared with other commonly used pretrained models. When combined with IFA loss, the model performance was significantly improved in and CheSS in 1% (*p* = 0.005), 10% (*p* = 0.011), and 50% (*p* < 0.001, CheSS) data fractions.

**Table 7:** Means and SDs of AUC experimented on 1%, 10%, and 50% data fractions, which were repeated 10 times with different random samples

	1%	10%	50%	100%
Scratch	0.562 ± 0.019^***^	0.765 ± 0.014^***^	0.807 ± 0.004^***^	0.811
ImageNet-1 K	0.765 ± 0.009^***^	0.839 ± 0.014	0.852 ± 0.005^***^	0.855
MoCo-ImageNet	0.769 ± 0.012^**^	0.840 ± 0.005	0.852 ± 0.004^**^	0.853
CXR-SimCLR	0.767 ± 0.013^***^	0.810 ± 0.004^***^	0.811 ± 0.003^***^	0.854
CheSS (CXR-MoCo)	0.770 ± 0.014^**^	0.833 ± 0.012^*^	0.841 ± 0.005^***^	0.843
SimCLR + IFA	0.763 ± 0.009^***^	0.804 ± 0.004^***^	0.809 ± 0.007^***^	0.865
MoCo + IFA	**0.789 ± 0.010**	**0.846 ± 0.003**	**0.862 ± 0.004**	**0.872**

The bold text indicates the best performance

The result of the full dataset was presented only with AUC. *SD*, standard deviation; *AUC*, area under receiver operating characteristics curve.

Finally, a pediatric pneumonia classification task was conducted. Table 8 lists the results of all experiments conducted. In the pediatric pneumonia classification task, CheSS, MoCo v2 pretrained with the CXR dataset, showed the best performance compared to the other models.

**Table 8:** Performance of pediatric pneumonia classification

	Accuracy	Recall	Precision	F1
Scratch	0.804^*^	0.805	0.806	0.805
ImageNet-1 K	0.816^***^	0.822	0.816	0.819
MoCo-ImageNet	0.819^***^	0.828	0.824	0.826
CXR-SimCLR	0.796^***^	0.803	0.804	0.803
CheSS (CXR-MoCo)	**0.854**	**0.853**	**0.848**	**0.851**
SimCLR + IFA	0.793^*^	0.797	0.801	0.799
MoCo + IFA	0.832^***^	0.832	0.836	0.834

The bold text indicates the best performance

Stuart–Maxwell test was conducted to compare scratch (randomly initialized), ImageNet, MoCo-ImageNet, CheSS, and our pretrained (intermediate feature approximation loss) models

^*^p < 0.05; ^**^p < 0.01; ^***^p < 0.001

### Object Detection Performance Evaluation

An object detection task of SIIM-FISABIO-RSNA COVID-19 object detection [32, 33] was conducted. As shown in Table 9, all pretrained models showed performance enhancement compared to the randomly initialized (scratch) model except NT-Xent pretrained model (SimCLR) in both R50-dilated-C5 and R50-C4 architecture.

**Table 9:** SIIM-FISABIO-RSNA COVID-19 lung opacity object detection

	R50-dilated-C5		R50-C4	
	*AP*_*50*_	*AP*_*COCO*_	*AP*_*50*_	*AP*_*COCO*_
Scratch	0.310	0.097	0.313	0.104
ImageNet-1 K	0.343	0.117	0.364	0.119
MoCo-ImageNet	0.348	0.119	0.362	0.119
CXR-SimCLR	0.321	0.102	0.319	0.104
CheSS (CXR-MoCo)	0.346	0.115	0.348	0.116
SimCLR + IFA	0.305	0.102	0.306	0.101
MoCo + IFA	0.361	0.117	0.350	0.110

Average precision (AP) at two different intersections of union (IoU) thresholds were evaluated (0.5, [0.5:0.95]).

### Medical Perceptual Loss Performance Evaluation

**Fig. 3:**
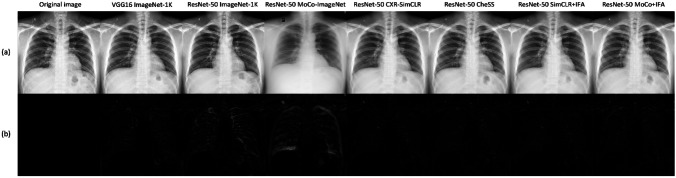
Example qualitative results of medical perceptual loss. The original image was projected into the corresponding latent feature using each pretrained network and subsequently reconstructed using StyleGAN2-ada through the process of GAN inversion. **a** Original image and reconstructed GAN inversion image. **b** Residual map between the original image and GAN inversion reconstructed image

As shown in Table 10, the medically pretrained models (SimCLR, MoCo v2 pretrained model using CXR dataset, and the models pretrained using IFA models) performed overall well on multiple quantitative metrics. NT-Xent loss pretrained model (SimCLR) significantly outperformed the other models in PSNR, SSIM, and RMSE (all *p* < 0.001). Medically pretrained models also showed good results in qualitative results (Fig. 3).

**Table 10:** Quantitative results of projecting original seed image into a latent feature with GAN inversion

	PSNR↑	SSIM↑	RMSE↓	LPIPS↓
VGG16 ImageNet	22.917^***^	0.679^***^	8.165^***^	0.162^***^
ResNet ImageNet-1 K	19.071^***^	0.589^***^	8.992^***^	0.240^***^
ResNet MoCo-ImageNet	18.735^***^	0.651^***^	9.257^***^	0.422^***^
ResNet CXR-SimCLR	**25.155**	**0.710**	**7.424**	0.211
ResNet CheSS (CXR-MoCo)	23.841^***^	0.691^***^	7.954^***^	0.193^***^
ResNet SimCLR + IFA	24.774^***^	0.705^***^	7.570^***^	0.214^***^
ResNet MoCo + IFA	23.815^***^	0.692^***^	7.970^***^	0.199^***^

The bold text indicates the best performance

*t*-test was conducted to compare VGG16-ImageNet-1 K, ResNet-ImageNet-1 K, ResNet-MoCo-ImageNet, ResNet-CheSS, and ResNet-our pretrained (intermediate feature approximation loss) models. *GAN* generative adversarial network, *PSNR* peak signal-to-noise ratio, *RMSE* root mean squared error, *SSIM* structural similarity index measure, *LPIPS* learned perceptual image patch similarity

^*^p < 0.05; ^**^p < 0.01; ^***^p < 0.001

## Discussion

In this study, we improved the baseline of an existing SSL network [18] by incorporating IFA loss with NT-Xent loss and InfoNCE loss on 4.8 M CXR images to capture the refined medical features better and revealed the results through the results of various downstream tasks. IFA loss combined models showed performance improvements in some medically important downstream tasks, such as overcoming data imbalances and data shortages. Although they did not achieve the best performance or significant performance improvements compared to CXR pretrained baselines (i.e., CXR-SimCLR, CheSS) in some tasks, IFA loss combined models still showed better results compared to commonly used pretrained models (i.e., ImageNet pretrained models). Some tasks, such as 6-class classification, may appear to show low performance. However, the performance is comparable to that of our previous publication and was fair enough given that the diagnostic performance of general practitioners or junior radiologists on CXR alone is known to be 40–70% [42, 43].

In convolutional neural networks (CNNs), low-level features, which are detected in the early layers of CNN, pertain to basic features such as edges, corners, and textures, whereas high-level features, which are detected in the deeper layers of CNN, pertain to more complex features such as object parts or the entire objects [44]. In medical imaging, low-level features can be referred to as lines and contours of each anatomical structure, and high-level features can be referred to as detailed anatomical variations. It is hypothesized that the CNN can learn common mid-level features (i.e., common anatomy) from various lower-level features by increasing the similarity between the intermediate feature maps of positive pairs. Furthermore, the CNN still can learn various anatomical variations (i.e., higher-level features) from common anatomy (i.e., common mid-level features). By combining the IFA loss with the NT-Xent loss or InfoNCE loss, the SSL network was also required to learn diverse high-level features to distinguish between negative pairs. Therefore, the IFA loss restricts the network from learning common features between positive pairs, while the InfoNCE loss encourages the network to learn diverse features between negative pairs, creating a complementary and mutually beneficial relationship.

Some attempts have been made to train pretrained models that are better suited to the task of medical image analysis in CXR. One research group [45] has attempted to collect pretrained models in their study. MoCo-CXR [46] demonstrates that self-supervised pretraining using CXR can improve the performance of deep learning in medical image classification learning. In addition, another research group [17] reported improvements in medical image analysis using pretraining on a large dataset of over 100 million medical images. Previous research from our group [18] also demonstrated performance improvements from the medical image pretraining. However, most studies have not included a sufficient number of downstream tasks that are critical and relevant in the field of medical image analysis. Our study extends upon previous research by adding pediatric medical image analysis, object detection, and capturing medical perceptual feature tasks, which have often been overlooked in the field. In addition, we have shown that combining IFA loss, which addresses the hard positive problems in contrastive learning, can improve the performance of medical contrastive networks. A recent study [26] has reported that addressing hard positives can improve medical image segmentation. It showed that using the cosine similarity of linear feature vectors from positive pairs can improve contrastive learning in medical image segmentation. Similarly, our IFA uses the cosine similarity, but we used the intermediate feature output of a network and showed the results in more diverse downstream tasks.

Our study has several strengths. First, the IFA loss proposed in this study can be easily implemented to enhance the performance of SSL in medical imaging. As demonstrated by the results, our model outperformed commonly used pretrained models in medical imaging by simply using the cosine similarity-based loss to approximate intermediate feature outputs of CNN. In addition, we conducted an ablation study to determine the optimal layer for feature approximation. Although using feature approximation could improve the performance of SSL regardless of the approximation layer, the results showed that block 3 provided the best overall performance, and thus, it was selected. Finally, we trained the network in a medically appropriate manner using a large amount of medical data, validated it through relevant and important medical tasks, and made the model publicly available.

However, our study also has several limitations. First, we were unable to make the data used in this study publicly available owing to the sensitive nature of medical data. As an alternative, we made this pretrained model publicly available. Second, some ablation studies, such as multiple feature layer approximation and multiple loss balancing, were not included owing to the limitations of resources. In addition, we did not experiment with over other distance-based losses, such as L1 or L2, for feature approximation owing to the limitations of resources. We preferred cosine similarity because it bounds the resulting loss between − 1 and 1, making it relatively easy to optimize early on [25] compared to other distance-based losses. Further studies will include thorough ablations studies. Although we trained a model on a large dataset of 4.8 M CXRs, the dataset was only from a single institution in a single country. Training on multiple data sources of multi-center and multi-continental data may improve the results. Finally, IFA only showed performance gains in some tasks in classification tasks, such as overcoming data imbalances and data shortages, but failed to show significant improvements in others. Many existing publications on the medically pretrained networks have only shown their superiority in classification tasks. It is important to use the appropriate pretrained weight depending on the target downstream task.

## Conclusion

We demonstrated decent performances and transferability of our contrastive learning with IFA loss. Our results demonstrate that the simple yet effective IFA loss can significantly enhance the performance of self-supervised networks in medical image analyses of CXRs. Furthermore, we have made our model publicly available to facilitate access and encourage further research and collaboration in the field.

### Supplementary Information

Below is the link to the electronic supplementary material.

Supplementary file1 (DOCX 626 KB)

## Data Availability

IFA weights are available at: https://github.com/chokyungjin/MI2RLNet_IFA.
