# Supplementary material for: Approximating Intermediate Feature Maps of Self-Supervised Convolution Neural Network to Learn Hard Positive Representations in Chest Radiography
Source: J Imaging Inform Med. 2024 Feb 21;37(4):1375–85. doi: 10.1007/s10278-024-01032-x (PMC11300846; doi:10.1007/s10278-024-01032-x)
Supplement: Supplementary file 1 — Supplementary file1 (DOCX 626 KB) [file 10278_2024_1032_MOESM1_ESM.docx]

**Appendix A. Implementation details for the main downstream task.**

*A.1 Multiclass classification using AMC CXR dataset*

The same number of images for each class was sampled for the undersampled dataset. Normal, nodule, and consolidation images were additionally sampled for the modified dataset, while the interstitial opacity images were simply duplicated because no additional data was available for interstitial opacity.

The implementation details are described as follows:

1) Preprocessing: The preprocessing was the same as that of the upstream task, and the dataset was split for training and validation at an 8:2 ratio.

2) Augmentation: The augmentation strategy was the same as that of the upstream task.

3) Training setting: For training, 1 GPU (NVIDIA Titan RTX 24GB) and a batch size of 48 were used. The network was trained using an SGD optimizer with a learning rate of 5e-2 using a weight decay of 1e-5.

*A.2* *Multilabel classification using the CheXpert dataset*

The implementation details are described as follows:

1) Preprocessing: Image preprocessing did not perform other intensity normalization since the original data included the histogram equalization.

2) Augmentation: Data augmentation was performed with *ShiftScaleRotate*, *MedianBlur, and GaussNoise*.

3) Training setting: Two GPUs (NVIDIA Titan RTX 24GB) and a batch size of 100 were used. SGD optimizer with an initial learning rate of 1e-3 and a weight decay of 1e-5 was used. The learning rate was reduced during the training following the ReduceLROn-Plateau schedule.

*A.3 Pediatric pneumonia classification using the Kermany, Glodbaum dataset*

The data augmentation method was used before the training phase to overcome data imbalance. After splitting the validation set, only the remaining training datasets were randomly augmented using rotation (±10°), scaling ($1\pm$0.2), and flipping (horizontal).

The implementation details are described as follows:

1) Preprocessing: The preprocessing was the same as that of the upstream task.

2) Augmentation: The augmentation strategy was the same as that of the upstream task.

3) Training setting: For training, 1 GPU (NVIDIA Titan RTX 24GB) and a batch size of 32 were used. In the training phase, SGD optimizer with a learning rate of 5e-4 and momentum of 0.9 was adopted.

*A.4* *COVID-19 lung opacity detection using SIIM-FISABIO-RSNA COVID-19 dataset*

The labels on the SIIM-FISABIO-RSNA COVID-19 detection public and personal test datasets were hidden, and scoring was only possible through the submission process. Accurate scores could not be obtained because the competition was designed to incorporate a single mAP score for both classification and detection tasks; thus, neither public nor private test datasets were used in this study.

The implementation details are described as follows:

1) Preprocessing: The preprocessing was the same as that of the upstream task. The SIIM-FISABIO-RSNA COVID-19 Detection train dataset contains 6,334 frontal CXR images in DICOM format, but only 6,030 images were used after data curation. It was curated to exclude data that was labeled opacity but did not have a bounding box. Only the SIIM-FISABIO-RSNA COVID-19 Detection train dataset was randomly split for our study, with 90% (5,426) used for the train set and 10% (604) used for the validation set. The CXRs consisted of both images with and without opacities because this study only focused on the detection portion of the challenge.

2) Augmentation: The augmentation was applied when learning the training dataset was largely divided into color (intensity) operation and Geometric operations. Color operations distorted color channels without affecting the locations of the bounding boxes, whereas geometric operations deformed the image, causing the location and size of the bounding box annotations to change [1]. *MotionBlur*, *MedianBlur,* CLAHE, *Sharpening*, *Embossing*, *RandBrightness, RandContrast, and Equalization* were selected for Intensity operation.

3) Training setting: For training with a backbone of R50-C4, 1 GPU (NVIDIA Tesla V100 32GB) and a batch size of 16 were used. The network was trained using an SGD optimizer with an initial learning rate of 2e-3 using a weight decay of 1e-5. For training with a backbone of R50-dilated-C5, 1 GPU (NVIDIA RTX 3090 24GB) and a batch size of 16 were used. The network was trained using an SGD optimizer with an initial learning rate of 3e-3 using a weight decay of 5e-4. In the training phase with both backbones, the learning rate was reduced during the training following the ReduceLROn-Plateau schedule.

*A.5 GAN inversion using VinDr-CXR Chest X-ray Abnormalities Detection dataset*

GAN inversion was performed using 10,606 CXRs with no finding class in the dataset.

The implementation details are described as follows:

1) Preprocessing: The preprocessing was the same as that of the upstream task, and the dataset was split for training and validation at a 9:1 ratio.

2) Augmentation: Data augmentation was performed with *ShiftScaleRotate*, *MedianBlur, and GaussNoise*.

3) Training setting: For GAN inversion, the style-based generative adversarial network [2] was trained. The model was used as the backbone for GAN inversion. For optimization, we used 1 GPU (NVIDIA RTX 3090 24GB) and 1000 steps. The network was trained using Adam optimizer with a learning rate of 2e-2.

**Appendix B. Qualitative results of the style-based generative adversarial network.**


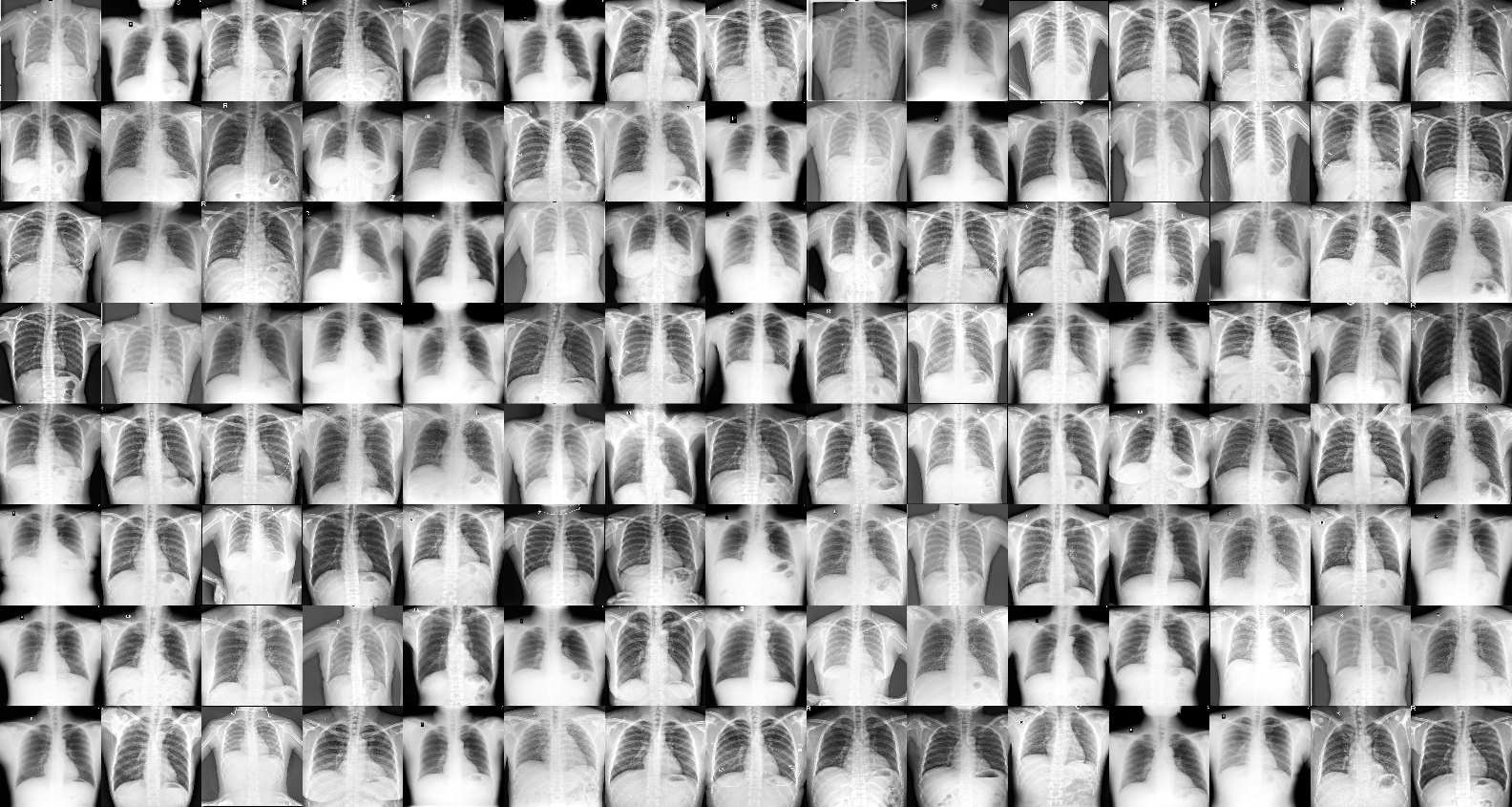


**Fig B.1.** Qualitative results of samples generated from the style-based generative adversarial network.

References

1. Zoph, B., et al. *Learning data augmentation strategies for object detection*. in *European conference on computer vision*. 2020. Springer.

2. Karras, T., et al., *Training generative adversarial networks with limited data.* Advances in Neural Information Processing Systems, 2020. **33**: p. 12104-12114.
